# Enhanced CellClassifier: a multi-class classification tool for microscopy images

**DOI:** 10.1186/1471-2105-11-30

**Published:** 2010-01-14

**Authors:** Benjamin Misselwitz, Gerhard Strittmatter, Balamurugan Periaswamy, Markus C Schlumberger, Samuel Rout, Peter Horvath, Karol Kozak, Wolf-Dietrich Hardt

**Affiliations:** 1 Institute of Microbiology, ETH Zurich, Wolfgang Pauli-Str. 10, 8093 Zürich, Switzerland; 2 Light Microscopy Centre, Institute of Biochemistry, ETH Zürich, Schafmattstr. 18, 8093 Zürich, Switzerland

## Abstract

**Background:**

Light microscopy is of central importance in cell biology. The recent introduction of automated high content screening has expanded this technology towards automation of experiments and performing large scale perturbation assays. Nevertheless, evaluation of microscopy data continues to be a bottleneck in many projects. Currently, among open source software, CellProfiler and its extension Analyst are widely used in automated image processing. Even though revolutionizing image analysis in current biology, some routine and many advanced tasks are either not supported or require programming skills of the researcher. This represents a significant obstacle in many biology laboratories.

**Results:**

We have developed a tool, Enhanced CellClassifier, which circumvents this obstacle. Enhanced CellClassifier starts from images analyzed by CellProfiler, and allows multi-class classification using a Support Vector Machine algorithm. Training of objects can be done by clicking directly "on the microscopy image" in several intuitive training modes. Many routine tasks like out-of focus exclusion and well summary are also supported. Classification results can be integrated with other object measurements including inter-object relationships. This makes a detailed interpretation of the image possible, allowing the differentiation of many complex phenotypes. For the generation of the output, image, well and plate data are dynamically extracted and summarized. The output can be generated as graphs, Excel-files, images with projections of the final analysis and exported as variables.

**Conclusion:**

Here we describe Enhanced CellClassifier which allows multiple class classification, elucidating complex phenotypes. Our tool is designed for the biologist who wants both, simple and flexible analysis of images without requiring programming skills. This should facilitate the implementation of automated high-content screening.

## Background

Automated analysis of microscopy images is of growing importance in many biological fields [1,2]. The improvements in microscopy and informatics hardware as well as the development of software tools have enabled ambitious experiments like genome scale RNAi screens, screening of large libraries of chemical compounds, etc. Image processing involves segmentation of the image into objects, in the biological setting usually nuclei and cells. Object attributes, for instance intensity, shape or texture can later be measured. For simple tasks, e.g. presence/absence of a color signal from a specific response reporter, a single object attribute is sufficient to distinguish biological phenotypes. However, biological questions often involve complex phenotypes that cannot be differentiated using a single object attribute. Changes in the cell organelle distribution or changes of the actin cytoskeleton are examples for this. Therefore, determination of such phenotypes makes the parallel evaluation of multiple object attributes necessary. This can be achieved by classification via machine learning approaches, for instance by specific supervised statistical pattern recognition algorithms. Supervised methods need training of objects by a user with prior knowledge; objects are thereby labeled to belong to one of several classes of phenotypes. A classification algorithm later utilizes the collected object attributes to identify a decision boundary between the phenotypes trained. An example for a commonly used classifier is the Support Vector Machine (SVM) algorithm [3].

In images of biological samples, typically objects with several complex phenotypes are simultaneously present on one image. Cell populations are inherently heterogeneous, for instance presenting themselves in different stages of their cell cycle. In addition, cells might react differently to a given experimental intervention. The combination of these phenotypes would make multi-class classification necessary for successful image analysis. In another common scenario, two independent objects might be identified on an image, for example the cell and a pathogen. Analysis might require first to classify one of these objects and later, classification information has to be collated with information about inter-object relationships. In conclusion, a tool which can handle multiple classes as well as inter-object relationships after classification is necessary. Enhanced CellClassifier is a software solution for such complex image analysis problems.

Commercial image analysis software has tremendously improved over the recent years. ArrayScan (Thermo Fisher Scientific) is one of the most popular programs. It is usually directly integrated with an automated screening microscope, enables image analysis with many features and handling of high content experiments. In this program, machine learning approaches are not supported but can be incorporated in connection with third party commercial data visualization and data mining software. Another program, Cellenger (Definiens), offers image analysis with great flexibility using pre-set modules and a powerful scripting language. An image browser, analysis modules and programming tools for image analysis including some machine learning algorithms are integrated in one single program that can also handle high content experiments. Limitations of some commercial programs include licensing fees and difficult customization, as well as lack of transparency of the analysis process and limited flexibility.

Among the currently available open source software resources, the Matlab based program CellProfiler [4] is popular and has been successfully used in many biological applications. It provides image segmentation and measurement routines as modules which can be flexibly combined. CellProfiler Analyst (CP Analyst) is a recently released CellProfiler extension which employs a gentle boosting algorithm for 2 class classification [5,6]. Even though the biological image analysis field has made tremendous progress because of the above programs, the user still faces limitations. Importantly, classification by CP Analyst is restricted to two classes. Moreover generation of a flexible output and solving of more complex image analysis tasks requires individual programming in addition to the usage of these tools. Other highly successful software projects focus on different aspects of image processing for instance subcellular localization of proteins [7-9], cell cycle phase identification [10], image segmentation [11], characterization of drugs based on cell phenotypic features [12,13], phenotypic changes after RNAi treatment [14,15], analysis of high content RNAi screening by time-lapse microscopy in a high throughput setting [16] or specific histopathology questions [17]; however, these approaches cannot easily be generalized. In a similar focused approach, the open source program ImageJ [18] has been used in combination with the image analysis program WEKA [19] to classify images of biological species [20]. Image analysis and classification can also be done with tools based on the open source platform R [21] including tools for analyzing RNAi-screens [22]. Some software packages focus on classification of whole images, not objects within images (for instance [23]). In addition, many of the above mentioned tools are used from a command line and might be useful mainly for bioinformatics experts but much less so for typical biological laboratories.

In a typical laboratory setting, experiments and microscopy are done by biologists without programming knowledge. Assays are typically performed in a 96 or 384-well format and 4-20 microscopy images are acquired in 1 to 3 or 4 channels per well. The task generally involves identifying the changes in cell phenotypes to evaluate the effect of the compound, condition or perturbation. When using the currently available software tools, in our experience four limitations are apparent: 1) Images belonging to the same well need to be summarized. 2) Out of focus images need to be excluded. 3) Several post-processing steps of the data need to be performed and 4) a comprehensive overview of data and an output in a human readable format needs to be generated. In CellProfiler, currently these tasks can only be accomplished by scripting. Since most biologists would need additional training before being able to write programs, tools for flexible post-processing analysis are required. For daily experiments with quickly changing conditions, flexibility of evaluation, ease of use and transparency of the evaluation process becomes very important.

In this publication we introduce Enhanced CellClassifier, a flexible and easy to use tool which allows classification as well as flexible post-processing that enables the user to evaluate many biological phenotypes. In the implementation section, we provide definition and description of important concept and terms, a detailed discussion of the SVM approach chosen by us and additional technical details. In the results section we present important features of our tool such as the graphical user interface, the different training methods and data integration and output generation. In addition we provide two biological case studies to illustrate the usage of our tool. Finally we compared our tool to another open source program, CP Analyst.

## Implementation

### Definition of important terms

#### Object

An object is an observation or an item on an image, defined and identified by an image analysis algorithm, for instance a recognized nucleus, cell or a spot.

#### Object attributes

Measurements of an object, for instance concerning its shape, intensity or texture; object attributes are often also referred to as object features.

#### Class

User defined phenotypic labels of an object are called classes. Classes might be mitotic, non-mitotic etc. During training the user assigns a class to an object.

#### Model

A set of instructions to predict the class of an object from object attributes. In Enhanced CellClassifier, a "model" can be trained, saved and reloaded, it contains the output of the SVM classifier, information about scaling of the data and the names of the object attributes.

#### 5-fold cross-validation accuracy

The data set is randomly divided into 5 equal parts. The classifier takes 80% of the data to calculate a model to predict the classes of the remaining 20% and to calculate the accuracy of this prediction. This is done five times and the accuracies are averaged.

#### Vector

In Enhanced CellClassifier vectors are user defined variables, important for summarizing and integrating data. A vector is binary (contains only the values zero or one) and has one type of object as its parent (for instance a vector might be derived from the nuclei of an image). Classification results, object attributes or inter-object relationships are translated into vectors for every image. The number of vectors that can be defined is not limited. Please refer to results section for an example.

#### Image, well and plate variables

User defined variables which integrate data and yield in a single value for an image, a well or a plate, respectively.

### Programming

Enhanced CellClassifier was programmed in Matlab. Graphical user interfaces were designed using the Matlab-feature GUIDE. Matlab was chosen because CellProfiler has also been written in Matlab; a good compatibility between both programs could thus be ensured. The program is easy to extend and to prototype.

### SVM classification

SVM is one of several supervised statistical pattern recognition algorithms. Such supervised machine learning algorithms classify objects of different classes according to their object attributes. In SVM, an object with n object attributes could be considered a point (vector) in an n-dimensional space of the object attributes. In a dataset consisting of two classes and appropriately chosen object attributes, the objects of each class might cluster together in this space. Therefore, in this n-dimensional space hyperplanes would exist, which separate the objects of the two different classes. In a simple case, the two classes could be distinguished by a linear separation and the hyperplane would lack any curves. A linear SVM algorithm would calculate the hyperplane separating these objects with the largest possible margin. The objects which are situated just at these margins and thus define the margins are called support vectors. If the dataset cannot be separated in a linear way, non-linear SVM-classifiers are used to calculate a curved hyperplane. For these calculations, the object attributes are mapped by a kernel function into a higher dimensional space in which the separation can be done linearly. Furthermore, in many practical examples, two classes cannot be separated perfectly; therefore a "soft margin" is introduced, where each misclassification yields in a "penalty" for the classifier, this penalty will be minimized by the algorithm (for a introduction see [24] and references therein). For multi-class classification, internally several hyperplanes are calculated to separate all possible pairs of classes; for three classes, 3 hyperplanes would be necessary (class 1 versus 2, 2 versus 3 and 1 versus 3).

In Enhanced CellClassifier, SVM-classification is done using libsvm [25]; this software package can handle multi-class classification and is integrated in Enhanced CellClassifier. Before training the data set is scaled, unknown to the user to ensure the best possible classification. We use a non-linear kernel (radial basis function (RBF)-kernel) to calculate models; other kernels are currently not supported. In the SVM window, the user can freely combine data from the current session and up to 10 previous sessions to form a set of training data and validation data. If a model is calculated, the accuracies of prediction for the training and validation data sets are calculated as well as the confusion matrix (matrix showing predicted classes versus trained classes).

In Enhanced CellClassifier, a separate window is dedicated for SVM training and adjustments. Our tool provides maximum flexibility in dividing all loaded objects into a training and a validation subpopulation, before calculating the model. The resulting model can thereby be tested against the current objects as well as objects from former training sessions. Optimization and adjustment of the critical parameters C (cost) and gamma of the RBF-kernel is supported [26], to optimize classification for different data sets. Enhanced CellClassifier also allows adjustment of the penalties for the different classes to ensure balanced classification of a heterogeneous data set. Histograms of the features of the trained cells can be visualized; apparently useless features can be excluded to save computation time.

While using SVM, Enhanced CellClassifier makes a powerful classifier available to the user; the approach worked better or as good as alternative classifiers in the biological examples 1 or 2, respectively (see below) and has been successfully applied on two unrelated microbiological problems (not shown). Moreover, reducing the numbers of classifiers to just one might avoid confusion for the novice user. However, our approach faces some potential limitations. First, in datasets containing an exceedingly large number of objects or a large number of object features, computational power for calculating models might become prohibitive. In this situation, for instance tree based classifiers or SVM using linear kernels might yield equivalent results in a shorter time frame. Enhanced CellClassifier users would need to apply feature selection techniques and exclude useless object features before training to overcome this potential problem. However, in our experience training time on normal desktop computers is usually within 1-5 seconds during normal usage; we tested datasets of up to 6000 cells and up to 400 features without training time exceeding 1 min. Second, optimal performance of the SVM classifier for some datasets (biological example 1) requires a grid search of the critical parameters C and gamma of the RBF-kernel which is supported by Enhanced CellClassifier. Otherwise poor classification of the training data set or overfitting, indicated by poor classification of the validation data set, might occur. In contrast, other classifiers either need no or considerably less parameter optimization. Finally, even though SVM with an RBF-kernel and correct settings can model every training data set accurately, it might predict a new data set not as accurately as other algorithms. Therefore, for some biological questions SVM using other non linear kernels, other classifiers or meta-classifiers might perform better than our approach even though we did not experience an example for this.

### Workflow in Enhanced CellClassifier

**Figure 1:**
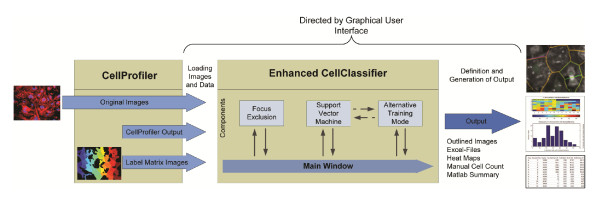
**Schematic view of the workflow in CellClassifier**. As an input, the tool needs the original microscopy images, as well as output files and label matrix images produced by CellProfiler. The interaction with the user happens mainly via the main window (for a screenshot see Figure 3). For special tasks like adaptation of the focus exclusion routine, Support Vector Machine model generation and alternative training as well as adjustment of the output and settings (not shown) more graphical user interfaces open. CellClassifier produces several different outputs: a) Outlined images with projections of the object borders, colors of the outlines thereby projecting classification or other object information (for examples see Figures 2 and 4). b) Excel-files with user defined information about the image, wells and the whole plate. c) Heat maps or other graphical summaries containing user defined overviews over a whole 96- or 384-well plate. d) Manual cell count, generating a well based summary of the classifications by the user and e) Matlab summaries, making export as Matlab variables possible.

Figure 1 shows an overview of the workflow in Enhanced CellClassifier. The input of our tool consists of CellProfiler output files (Matlab files containing object data, for instance measurements of identified cells), CellProfiler generated label matrix images (images showing the borders of the objects) and the original microscopy images. The output could be graphs, images demonstrating the analysis, Matlab data and Excel-files.

### Integration with other programs

Enhanced CellClassifier can load CellProfiler data; the output of other image analysis programs is currently not supported. Data from trained Enhanced CellClassifier objects including object attributes and their classification can be saved as '.arff' files and imported into the open source program WEKA (Waikato environment for knowledge analysis) [27] for further analysis of the data with different classifiers. In addition, the data can be imported into the recently released open source program HCDC-KNIME http://hcdc.ethz.ch, [28]); this workflow based system enables linking Enhanced CellClassifier output data with the original images, experimental data (for instance RNAi-data) and further advanced bioinformatics analysis.

### Flexible and automated focus analysis

Autofocus problems are virtually unavoidable when working with automated microscopes. Therefore, image series are likely to include a small number of images which are out of focus and therefore unsuitable for analysis. It is desirable to exclude those images or at least identify such problems. We use the CellProfiler module "MeasureImageGranularity" which performs several rounds of image erosion followed by image reconstruction and measures the difference of the mean image intensity after each round [29,30]. For images with many objects and high contrast these values will be high whereas out of focus images or images with fewer objects yield lower values. In a designated window, we plot the number of nuclei (or another user-selected object) against the image granularity. The user trains images as being in focus or out of focus; finally a line is adjusted separating both groups of images in this plot. If the user wants, out of focus images will be excluded; in that case they will never be presented for training and are not summarized when the final output is generated. In the two biological examples provided, focus exclusion has not been necessary.

### Demonstration and manual

For the demonstration of our program, two different biological examples (HGF-ruffling and *Salmonella *docking) were chosen; a brief tutorial guides step by step through the demonstration. The examples are integrated in the program and can immediately be used after starting Enhanced CellClassifier. Our tool is accompanied by an extensive manual covering all important aspects of Enhanced CellClassifier.

The experiments for the two biological examples are briefly described within the text, further experimental details and details of the CellProfiler analysis pipelines used are available upon request.

The program can be downloaded at: http://www.micro.biol.ethz.ch/downloads

## Results and Discussion

### Program Feature 1: Training and interactive decision making

Enhanced CellClassifier is a novel application which efficiently integrates image analysis results from the open source program CellProfiler [4] with SVM classification algorithms [25]. Multi-class classification is a distinguishing feature of Enhanced CellClassifier. The current version of Enhanced CellClassifier supports five classes; a case study involving 3 classes is given in biological example 1 below: Hepatocyte growth factor induced ruffling of HeLa cells. Enhanced CellClassifier facilitates image display in a browser which supports three channels, scaling, zooming, and image navigation. The images are randomly selected from user defined image groups which correspond to the wells from which the images are derived. The class of an object is directly shown on the image; the color of the outline of the object thereby indicates the class. Both, display of images and the presentation of objects can be customized.

During supervised learning, the user labels the objects; thereby attributing a class to them (a cell for instance could be mitotic or non-mitotic). These objects (for instance cells) had first been identified and measured by CellProfiler; object attributes (object features) are thereby extracted (for instance mean intensity of the cell in the actin channel). Thereby, a data set of trained cells containing object attributes and the class label is assembled. The algorithm of the classifier then calculates a set of instructions to predict the object labels from the measured object attributes. Several strategies to achieve this have been proposed and successfully applied including decision tree based, Bayesian and nearest neighbor classifiers, neuronal networks, perceptrons and Support Vector Machines (SVM). Enhanced CellClassifier uses an SVM algorithm with a radial basis function (RBF) kernel for training; the open source program libsvm [25] is integrated in our tool and is exclusively used for these calculations. A detailed discussion of SVM and machine learning is given in the implementation section.

**Figure 2:**
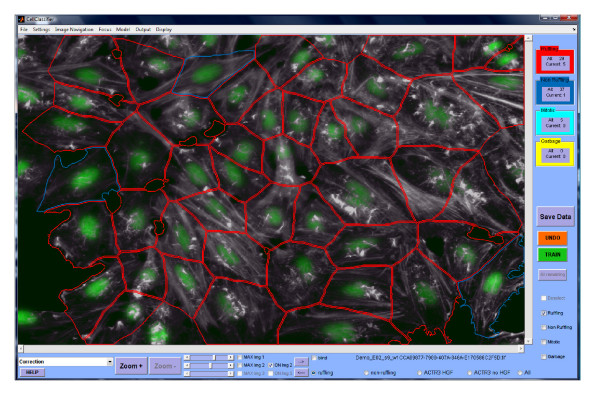
**CellClassifier program: Screenshot of the main window**. The program is currently in the correction mode, which shows the predictions of the current mode, allowing the user to correct. Red outlines: ruffling cells, blue outlines: non-ruffling cells (outlines exaggerated for clarity).

In Enhanced CellClassifier, training is done by direct clicking on the respective object on an image (Figure 2). Training strategies might critically influence the classification process; Enhanced CellClassifier offers four intuitive training modes. Training in the exploratory mode ("default") provides maximum flexibility to the user to freely select any object. However, in this mode the user might avoid frequent borderline phenotypes. Therefore, a second mode, "Random", exists. This is a forced choice mode; the user is required to decide about the phenotype of randomly selected objects from a randomly selected image. This training mode thereby avoids any selection bias by the user. At any time point training can be interrupted for calculation of a SVM model.

In a later stage of the training process, the user might want to refine a preliminary model. Training more objects would obviously be useful. However, a more efficient strategy would to be to limit training to objects which had been difficult to classify for the algorithm. The predictions for these objects will either be incorrect or just correct; the objects are located at the decision boundaries for the classifier. Therefore, in a third training mode, the "correction" mode, predictions for all objects will be displayed on the image. Only objects corrected by the user will be memorized. Adding these borderline objects to the data set can greatly improve the model. Finally, in a fourth mode, the "decision boundaries" mode, only objects for which the predictions are closest to the decision boundaries of the current model are presented; these objects are also most valuable for further refinement. The user can freely switch between training modes; moreover, training can be performed in an "informed" or a "blinded" fashion, either displaying the image filenames or not.

Presentation of objects within the original images directly illustrates the biological process, image segmentation and performance of the classifier to the user. It might enhance training accuracy, since the context of each object can be taken into account by an experienced biologist. On the other hand, the context might result in a training bias and image based training might cause under-representation of objects from images with high cell densities. Therefore, training can also be conducted in another window; here, 10 individual objects from up to 10 different images from the 96- or 384-well plate will be displayed. 8 of those objects are selected to be close to the decision boundaries of the current model, the remaining 2 illustrate the positive and negative phenotype. This mode avoids any training bias and might enhance the efficiency of the training process by selecting the most interesting objects from the whole plate. A similar training mode has recently been described [6].

### Program feature 2: data integration

Enhanced CellClassifier facilitates the integration of classification information with other CellProfiler data. During image analysis by CellProfiler inter-object information can be calculated. For instance, two independent object types can be related to each other if they overlap (for instance cells and "spots", see below); one object will be labeled as the child of the other. With a different module, neighborhood information for objects of the same kind can be calculated. However, in CellProfiler this information can only be utilized after individual programming.

Enhanced CellClassifier allows the user to define internal representations of the objects which we call "vectors". A vector is binary and will be calculated for each image; it contains as many numbers as objects of the specified kind. Classification, measurement and inter-object information can all be translated into binary vector information. Since one can generate new vectors from existing vectors using logical operations, the user is now able to define any subgroup of objects desired. For example: If an image contains 5 cells, of which 1 and 3 are mitotic, the vector for mitotic cells for this image would be 1, 0, 1, 0, 0. If cells 1, 4 and 5 are calculated to be infected by a pathogen, the vector for infected cells would be 1, 0, 0, 1, 1. The vector infected mitotic cells would be 1, 0, 0, 0, 0, infected non-mitotic cells 0, 0, 0, 1, 1 and so on. This vector concept enables the user to handle cases combining classification and inter-object relationships or other object properties which would otherwise only possible by scripting. Feature integration is illustrated below in the biological example 2, *Salmonella-*docking onto HeLa cells.

### Program feature 3: Dynamic data extraction

To ensure the greatest possible flexibility three further internal representations can be defined by the user: "image variables ", "well variables " and "plate variables". Image variables comprise just one number for each image, for instance "number nuclei", "number infected cells" or "percent infected cells". They are in most situations calculated from vectors; however, Enhanced CellClassifier also allows importing CellProfiler data directly, for instance threshold information. Well variables are summaries of the image variables of one well. Well variables can also be the result of a calculation, for instance the normalization of the number of docked or ruffling cells by the total number of nuclei in this well. Plate variables are summaries of variables from wells chosen by the user. They are especially useful for normalizing all data on a plate or for bringing internal controls prominently to the attention of the user. All variables are defined via a graphical user interface where predefined choices avoid "impossible" settings.

### Program feature 4: Flexible output options

**Figure 3:**
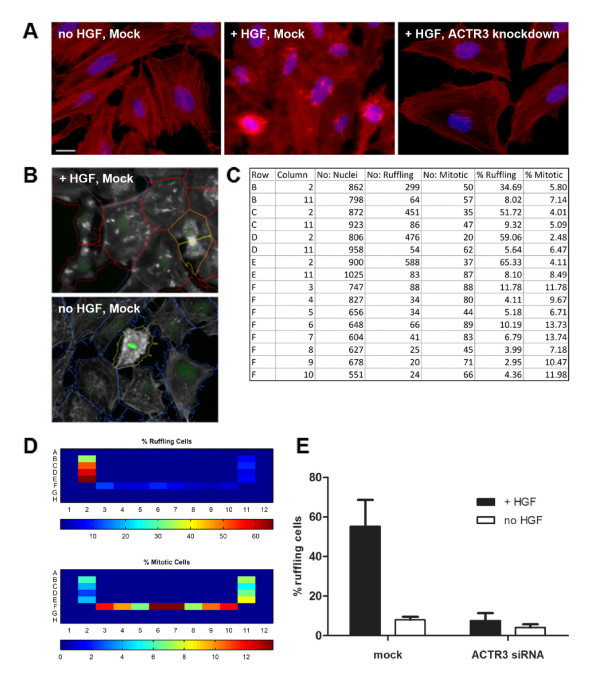
**HGF-induced ruffling**. **A**: HeLa-cells were pre-treated either under Mock-conditions or with an siRNA directed against the mRNA of the ACTR3 component of the Arp2/3 complex. Later, HGF was added, where indicated. Red: Actin, Blue: Nuclei. Scale bar: 20 μm. **B: **After image segmentation and measurement by CellProfiler, a model was trained in CellClassifier, outlined images showing predictions were produced. Grey: actin, green: nuclei, red outlines: ruffling cells, blue outlines: non-ruffling cells, yellow outlines: mitotic cells. **C**: Part of the Excel-sheet generated by CellClassifier. **D**: Illustration of CellClassifier output, left: heat maps. Positions of mock treated wells +HGF: B02-E02, no HGF: B11-E11. siRNAs against ACTR3 mRNA were positioned at F03-F06 (+HGF) and F07-F11 (no HGF). Only wells of interest were imaged. **E**: Summary of the experiment generated outside CellClassifier. Cells were pre-treated with siRNA and incubated with HGF as indicated. Each bar shows the median and standard deviation of 4 wells. With ACTR3 each well was treated with a different siRNA against ACTR3, yielding virtually identical effects.

**Figure 4:**
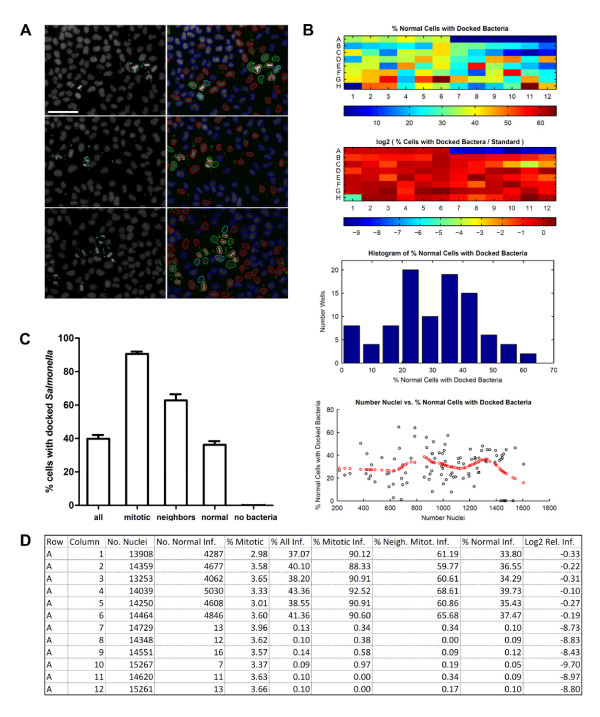
**Feature integration by CellClassifier**. **A**: *Salmonella *(shown in green) preferentially dock onto mitotic cells (nuclei shown in grey). Segmentation and measurements of image were done using CellProfiler: First both, nuclei and *Salmonella*, were identified as independent objects. Cell objects were generated by expansion with reference to the nucleus. Inter-object relationships between *Salmonella*-spots and cells, as well as between neighboring nuclei were calculated. In CellClassifier a model was trained to distinguish mitotic cells from non-mitotic cells. Cells with at least 1 associated spot were considered infected. Using the feature integration properties of CellClassifier, 6 population of nuclei (6 vectors) were calculated and exported to Excel and as outlined image: Infected mitotic cells (orange outline), non infected mitotic cells (pink, very rare therefore not shown), cell with mitotic neighbor, infected (green), mitotic neighbor, non-infected (cyan), normal cell, infected (red), non-infected (blue). Scale bar: 100 μm. **B**: Illustration of CellClassifier graphical output (heat maps). The experiment was done in a 96-well plate. Row A represents mock conditions (A01-A06) and no bacteria (A07-A12). The diagram in the upper left shows percentage of normal cells infected; the diagram in the upper right cell shows the log2 of the percentage of infected normal cells, normalized to a standard (G01-G12). Lower left: histogram of percent infected cells in the whole 96-well plate. Lower right: % normal cells infected plotted as a function of number nuclei. The red circles represent a trend analysis determined by a sliding window. In this plot, no gross trends are obvious. **C**: Summary of the output done outside CellClassifier, showing the preference of *Salmonella *docking for mitotic cells. **D**: Part of the Excel-file generated by CellClassifier.

Most important for the user is the summary of the whole experiment in a comprehensible and human readable format. Our program generates four different kinds of output data: outlined images, Excel-files, graphical summaries and a Matlab readable output. Outlined images visualize a vector or the result of the classification for a given image; if for instance the user wanted to visualize the vector "mitotic cells" using a yellow color, for all objects for which the vector had been positive (i.e. all mitotic cells) the outlines would be stained yellow (for examples see Figures 3, 4). Outlined images allow for a visual control of the final analysis and documentation. Excel-data are probably the most popular data format for biologists; all image, well and plate variables are automatically exported to an Excel-sheet. Well variables from the whole plate can be visualized as heat maps, histograms or scatter plots. They allow a quick overview over the whole experiment. When doing larger experiments, the user might want to do further customized analysis. Therefore, image and well variables of interest can be exported in a Matlab readable format.

Enhanced CellClassifier supports automatic processing of multiple plates. To allow for the analysis of a high content screen, the Enhanced CellClassifier output can be imported into the open source program HCDC-KNIME which enables the analysis of large experiments as well as integration with the original images, experimental data and further advanced bioinformatics analysis.

### Biological example 1: Hepatocyte growth factor induced ruffling

We chose ruffling of cells in response to hepatocyte growth factor (HGF) as an example for automated identification of complex phenotypes. HGF is a cytokine which can stimulate cell motility, proliferation and morphogenesis. A visible sign of HGF-activity is the appearance of pronounced "dorsal" ruffles on the surface of the cell [31]. Ruffles are driven by rapid actin polymerization. In this context, the master regulator of actin polymerization Arp2/3 with its central component ACTR3 is known to play a major role. The intracellular signaling from HGF-receptor leading to ruffling is currently a subject of intense research [32].

We established an image based ruffling assay using HeLa cells (Figure 3). HeLa cells were incubated for 5 minutes with HGF at a final concentration of 100 ng/ml, fixed and stained with 4', 6-diamidino-2-phenylindole (DAPI), for visualization of nuclei and tetramethyl rhodamine iso-thiocyanate (TRITC)-phalloidin for staining of the actin cytoskeleton. Where indicated, cells had been incubated with Lipofectamine 2000 and 20 nM of an siRNA directed against the mRNA of ACTR3 for down regulation of this protein prior to the assay. 20 images were acquired per well with an Image Xpress microscope (Molecular devices) using a 20×-objective. In the microscopy images, nuclei and cells were identified in the DAPI and the actin channel, respectively using established CellProfiler modules.

Cells and nuclei were subsequently measured in both channels using CellProfiler tools for measurements of object texture and intensity. In brief, CellProfiler texture measurements include Haralick features, comprising a set of statistical calculations derived from the grey level co-occurrence matrix of an object [33] and Gabor features, obtained after applying Gabor filters [34] in the x and y direction. Intensity measurements include the minimum, maximum, median and mean pixel intensities over an object and its edge regions, respectively. Both, intensity and texture measurements were performed for the actin- and DAPI-channel of the image for the region of the nucleus and the cell.

In order to improve the performance of the classifier, customized CellProfiler modules were developed. Our modules take advantage of the high difference in the intensity of a ruffle in the actin channel compared to the remainder of the cell and their distinctive compact shape. In brief, in one strategy we determined the regions of the cell with the brightest intensities, either by applying a fixed circular mask or by thresholding using the Otsu algorithm [35]. Subsequently, features describing the contrast between the brightest area and the remaining area of the cell were extracted [36]. In an additional approach, we took advantage of the fact that the area of ruffles within a cell consisted usually of the 5% brightest pixels within a cell. The shape of the thus identified regions was measured (solidity, eccentricity) as well as the contrast (difference, z-score) of the potential ruffle relative to the remainder of the cell.

However, no single feature could clearly distinguish ruffling from non-ruffling cells (not shown). This was not entirely unexpected, since changes in actin polymerization also happen during normal cellular life, for instance at the entry into mitosis. Therefore this problem required to identify three different cell types: ruffling, non-ruffling and mitotic cells. This task could conveniently be achieved using Enhanced CellClassifier.

For identifying dorsal ruffles on HGF-treated cells, objects were trained in the "default" and "random" training mode. After training a preliminary SVM model, incorrectly classified cells were corrected in the "correction" mode, yielding a final data set of 782 objects. After a grid search of the parameters C and gamma for the RBF-kernel, the 5-fold cross-validation accuracy was 87.7%. This slightly less than optimal performance is most likely due to the presence of weakly ruffling cells with a borderline phenotype which are difficult to classify, even for a human observer. In agreement with this interpretation, a detailed look on the confusion matrix of the 5-fold-cross-validation procedure showed, that mitotic and non-ruffling cells were mainly correctly predicted (94% and 91%, respectively), in contrast to ruffling cells (77%) which were frequently misclassified as non-ruffling (22%). In further tests with the same images, a dataset containing 340 objects with exclusively strong phenotypes was classified almost perfectly (5-fold cross-validation accuracy 95%), while another dataset from the same image set containing 700 objects trained in a strictly random and blinded manner yielded a 5-fold cross-validation accuracy of 85.7%.

To allow for experimental comparison of the performance of different classifiers, our dataset (782 objects) was exported to the open source program WEKA. We tested a large set of classifiers of which only few algorithms approached the accuracy of SVM with an RBF-kernel (additional file 1). From these tests we conclude, that for this dataset the performance of libsvm with an RBF-kernel and our settings cannot easily be outperformed by other algorithms.

When the different object features were ranked by WEKA for their ability to distinguish ruffling from non-ruffling cells using different algorithms, for instance SVM attribute selection [37], the object attributes describing texture in the actin channel consistently ranked best followed by our customized object attributes. To determine the relationship between the number of available object attributes and the 5-fold cross-validation accuracy, we systematically tested the performance of our classifier using increasing numbers of object attributes. We started with one attribute and added more attributes in the order suggested by the SVM attribute selection algorithm and optimized the kernel parameters C and gamma. A set of 28 object attributes performed best, achieving a 5-fold cross-validation accuracy of 89%, thereby marginally exceeding the cross-validation accuracy of the whole set of object attributes. Object attribute selection (restricting training to a subset of object attributes) has the additional advantage of decreasing training time. Nevertheless, a model for this dataset could be calculated in only 2 seconds on a desktop computer, therefore no further attempts were made. Object attribute selection algorithms are currently not supported by Enhanced CellClassifier but will be the scope of future developments.

In summary, Enhanced CellClassifier could identify mitotic and non-ruffling cells with high and ruffling cells with moderate accuracy. Using our tool in the "correction" training mode, the biologist can directly visualize the predictions of the model on different images; this increases the confidence of the researcher to the analysis algorithm. Subsequently, the model was applied to the complete dataset of the biological experiment. As shown in Figure 3, frequent ruffling was observed under control conditions; in contrast, without HGF, only background ruffling was observed. Moreover, elimination of a critical component of the cascade leading from HGF to actin polymerization also reduced ruffling: After siRNA mediated knockdown of the ACTR3-component of the Arp2/3 complex, ruffling was reduced to background. Therefore, Enhanced CellClassifier can automatically analyze HGF-induced ruffling. This could be useful for future identification of new intracellular proteins important for ruffling.

### Biological example 2: Docking of Salmonella onto HeLa cells

*Salmonella *Typhimurium is an important food borne pathogen causing diarrhea and rarely systemic disease. Central to the pathogenesis by *Salmonella *is its ability to invade epithelial cells [38,39]. Docking onto cells is the first crucial step of the infection by *Salmonella*. This process can be studied in tissue culture: Cells were incubated with the non-invasive *Salmonella *Typhimurium strain M566 (SL1344 ΔSipA, ΔSopBEE2, [40]) for 6 minutes, washed and fixed. Nuclei were visualized using DAPI, *Salmonella *by indirect immunofluorescence in the green channel using a rabbit antibody directed against the O-side chain of LPS (Difco). 4 images per well were acquired with a 4×-objective. Using CellProfiler modules, nuclei could be identified in the DAPI-images. Cells were defined by expansion of the area of the nucleus. Infectious "spots" representing single bacteria or a small number of *Salmonella *cells were identified as independent objects in the green channel. During CellProfiler analysis inter-object data were collected: the relationship of spots and cells was determined using the CellProfiler module "relate": any spot overlapping with a cell was labeled the child of this cell. In addition, neighborhood information of different cells was also calculated.

When looking at the microscopy images, a striking preference of *Salmonella *for mitotic cells was observed (Figure 4). *Salmonella *were also enriched at cells adjacent to mitotic cells. Therefore, when investigating the docking process, the researcher would like to quantify docking properties for 3 types of cells: mitotic cells, neighbors of mitotic cells and non-mitotic cells.

Nuclei of mitotic cells can easily be recognized in the DAPI-channel by the human observer. However, for automated analysis more than one object attribute was necessary (not shown). Therefore, the final analysis was done using Enhanced CellClassifier. Two classes were defined: mitotic and non-mitotic nuclei and trained first in the "default" training mode, followed by a refinement of a model in the "correction" training mode. The final data set contained 2001 objects from samples of forty 96-well plates of 2 independent experiments.

Object attributes, measured by CellProfiler available to the classifier were intensity and texture measurements of the nuclei in the DAPI-channel (for explanation see biological example 1). The object attributes ranking best according to their ability to distinguish between classes [37] included intensity measurements followed by Gabor and Haralick features (not shown). The combination of these object attributes enabled the SVM-algorithm to reliably distinguish between mitotic and non-mitotic nuclei with a 5-fold cross-validation accuracy of 96.0%. Using a large data set including 2000 cells was not necessary to achieve reliable discrimination between the classes, since 5-fold cross-validation accuracies above 93% were consistently achieved with random samples as small as 250 objects. Nevertheless, a larger data set did not require extensive computational power, since calculations needed only 2.5 seconds on a desktop computer.

Other classifiers performed equally well on this data set: after exporting the training data set to WEKA, 5-fold cross-validation accuracies ranging from 94% to 97% were obtained with the 20 algorithms tested (additional file 1).

For the summary of the data 3 vectors were calculated: one with information about the cell cycle (mitotic, non mitotic), the second with information about neighborhood to mitotic cells and a third with information about associated spots. Combining these three vectors yielded all the desired subtypes of cells (Figure 4). As shown in Figure 4C, mitotic cells, neighbors of mitotic cells and normal cells differ greatly in the percentage of docked *Salmonella*. To the best of our knowledge, this is the first demonstration of the docking preference of *Salmonella *to mitotic cells; however, the biological basis for this interesting phenomenon remains elusive. In any case, to investigate the docking process independently from the cell cycle, the user can now concentrate on the purged cell population.

### Experimental comparison of Enhanced CellClassifier with CP Analyst

We wanted to compare our new tool with existing software; among the available open-source software only the program CellProfiler Analyst (CP Analyst) was developed with a similar scope as our tool: flexible image analysis using machine learning algorithms for biologists without the need for scripting.

We compared several aspects of the two programs including the scope of the classifier, the training process, and the user interface and export options. Importantly, CP Analyst is limited to 2 class classification where as Enhanced CellClassifier can manage up to 5 different classes. This is a limitation for the analysis of many complex biological phenotypes. For the training process, both, CP Analyst and Enhanced CellClassifier provide innovative methods. In CP Analyst, the algorithm selects for presentation of an adjustable number of objects, either randomly or of the "positive" or "negative" phenotype; these objects are chosen to be close to the decision boundaries of the current model and can quickly improve the current model. The different training options of our tool have been described above. In CP Analyst, the user interface is less friendly and offers very little flexibility. The objects are presented to the user as little image snippets which have to be sorted into a bin of positive and negative objects. Therefore, visual judgment of the object phenotypes becomes extremely difficult. In contrast, Enhanced CellClassifier offers many options for image presentation in order to ease visual inspection and training. Furthermore, Enhanced CellClassifier provides a visual feedback of the current model on the whole image which allows for immediate evaluation of the performance of the current model. In contrast, CP Analyst lacks such a feature. Finally, CP Analyst uses a MySQL database for data retrieval which facilitates quick summarization of data. However, output options were severely limited; for example, the results of the 2-class classification cannot be integrated with other object information. In addition, customization or well-based data summary were not supported. In comparison, Enhanced CellClassifier has a dynamic way of integrating results with maximum flexibility. This allows the user to define an output with almost unlimited options (details discussed above).

For experimental comparison, we chose our biological examples mentioned above. For biological example 1, classification had to be simplified since CP Analyst only supports two classes; mitotic cells therefore could not be simultaneously identified. However, the program could clearly distinguish ruffling and non-ruffling cells and recognized the phenotypes of the RNAis tested in this experiment (not shown). In biological example 2, the program could learn to distinguish mitotic from non-mitotic cells, however, the differential analysis we did with Enhanced CellClassifier to measure the percentage of infected cells for mitotic cells, its neighbors and interphase cells were not possible with CP Analyst.

In summary, while classification of biological objects is also possible in CP Analyst, the user is restricted to two-class classification and an inflexible display and output which only provides most basic analysis options. Most likely, these problems will be solved in the next version of this software, Classifier 2.0, which is not available for windows yet.

## Conclusion

In summary, Enhanced CellClassifier is a user-friendly and intuitive tool which allows multi-class classification of biological objects in many intuitive and performance enhancing training modes. For feature integration, classification information can subsequently be combined with data about inter-object relationships and object measurements, greatly enhancing the evaluation options. Further useful features are focus exclusion, well summary, and specific calculation and normalization options. The output function which can be defined by the user within broad ranges should cover many needs of image analysis in a biological setting. Our tool greatly facilitates image analysis for biologists without requiring programming skills.

## Availability and requirements

Project name: Enhanced CellClassifier

Web page: http://www.micro.biol.ethz.ch/downloads

Operating system: Platform independent

Programming language: Matlab

Other requirements: Matlab 7, full version, release 2008a or later

License: GNU GPL

## List of abbreviations

DAPI: 4',6-diamidino-2-phenylindole; HGF: hepatocyte growth factor; RBF: radial basis function; SVM: support vector machine; TRITC: Tetramethyl rhodamine iso-thiocyanate; WEKA: Waikato environment for knowledge analysis

## Competing interests

The authors declare that they have no competing interests.

## Authors' contributions

BM designed and developed the program, did the *Salmonella *experiments and wrote the paper. GS designed and performed the HGF experiments. BP participated in interpretation of the data, and writing of the manuscript. MS, PH, KK participated in interpretation of the data. SR performed the *Salmonella *experiments. WDH designed the research and participated in interpretation of the data and writing of the manuscript. All authors read and approved the final version of the manuscript.

## Supplementary Material

Additional file 1 **Comparison of SVM with RBF kernel with other classifiers**. Classifiers were tested using WEKA [19]. Meta-classifiers were tested in combination with the classifiers performing best when tested alone including Random Forrest, J48, Simple Logistic and Decision stump. Please refer to the documentation of the WEKA program for a detailed description of the classifiers and respective references. Click here for file
