# Supplementary material for: Enhanced CellClassifier: a multi-class classification tool for microscopy images
Source: BMC Bioinformatics. 2010 Jan 14;11:30. doi: 10.1186/1471-2105-11-30 (PMC2821321; doi:10.1186/1471-2105-11-30)
Supplement: Additional file 1 — Comparison of SVM with RBF kernel with other classifiers. Classifiers were tested using WEKA [19]. Meta-classifiers were tested in combination with the classifiers performing best when tested alone including Random Forrest, J48, Simple Logistic and Decision stump. Please refer to the documentation of the WEKA program for a detailed description of the classifiers and respective references. [file 1471-2105-11-30-S1.DOCX]

| Classifier | 5-fold cross-validation accuracy  For biological example 1 -  HGF induced ruffling | 5-fold cross-validation accuracy  For biological example 2 -  Docking of *Salmonella* |
| --- | --- | --- |
| CellClassifier  - libsvm with RBF-kernel | 87.7% | 96.0% |
| **Rule based classifiers** | | |
| Zero R | 59.6% | 70% |
| One R | 68.1% | 94.3% |
| JRip | 83.6% | 94.8% |
| **Tree based classifiers** | | |
| Decision stump | 67.3% | 94.4% |
| J48 | 79.2% | 95.7% |
| Random Forest | 85.5% | 97.2% |
| Random Tree | 76.9% | 95.6% |
| **Bayesian classifiers** | | |
| Bayes Net | 83.6% | 95.0% |
| Naive Bayes | 82.2% | 94.8% |
| **Lazy classifiers** | | |
| IB1 | 79% | 94.7% |
| K* | 76.6% | 94.2% |
| **Functions** | | |
| Logistic | 82.9% | 96.0% |
| Simple Logistic | 86.4% | 95.6% |
| Multilayer Perceptron | 86.1% | 96.3% |
| Decision Table | 77.1% | 95.3% |
| SVM (SMO) polynomial kernel | 85.4% | 96.2% |
| **Meta-classifiers** | | |
| AdaBoostM1 | 86.1% | 96.9% |
| MultiBoost AB | 85.3% | 96.8% |
| Bagging | 85.4% | 96.8% |
